# Transarterial N-butyl Cyanoacrylate Embolization of a Pediatric Pial Arteriovenous Fistula Using a Distal Access Intermediate Catheter for Microcatheter Support: A Case Report

**DOI:** 10.7759/cureus.102670

**Published:** 2026-01-30

**Authors:** Genki Ikuta, Yushin Takemoto, Ryosuke Mori, Airi Miyazaki, Tatemi Todaka

**Affiliations:** 1 Neurosurgery, Japanese Red Cross Kumamoto Hospital, Kumamoto, JPN

**Keywords:** embolization, endovascular treatment, intermediate catheter, pial arteriovenous fistula, vecta 46

## Abstract

Pial arteriovenous fistulas (pAVFs) are rare, high-flow cerebrovascular malformations characterized by direct arteriovenous shunting without an intervening nidus, and treatment selection depends on angioarchitecture, clinical severity, and institutional expertise. A seven-year-old boy presented with intracerebral hemorrhage caused by a right temporal pAVF supplied by the superior trunk of the middle cerebral artery, with cortical venous drainage into a vein anatomically consistent with the vein of Labbé. Hematoma evacuation and decompressive craniectomy were performed, followed by elective transarterial embolization. A distal access intermediate catheter was advanced to provide distal support and to allow contrast injection while a microcatheter was in place, and n-butyl cyanoacrylate (NBCA) was injected through the microcatheter positioned adjacent to the fistulous point. Final angiography demonstrated complete shunt disconnection with preservation of the cortical venous drainage pathway. This case describes a catheter support strategy facilitating microcatheter-based NBCA delivery without adjunctive balloon or coil assistance in a pediatric high-flow pAVF.

## Introduction

Pial arteriovenous fistulas (pAVFs) are rare cerebrovascular malformations consisting of one or more direct arterial connections to a single draining vein without a nidus, often resulting in high-flow shunting and venous varix formation [1-3]. In older children, pAVFs may present with intracerebral hemorrhage and require prompt intervention to prevent neurological deterioration [1].

Management options include microsurgical disconnection and endovascular embolization. Neither approach is universally preferred; treatment selection depends on lesion location, angioarchitecture, and operator experience [2,4]. A key technical challenge in endovascular treatment of high-flow pAVFs is achieving stable distal access and adequate visualization to enable controlled liquid embolic delivery while minimizing the risks of reflux, distal migration, or inadvertent venous occlusion [3,4].

This case report describes a pediatric hemorrhagic pAVF treated with transarterial n-butyl cyanoacrylate (NBCA) embolization using a distal access intermediate catheter to support microcatheter positioning and permit contrast injection with the microcatheter in place. The report focuses on practical considerations of access construction and embolic delivery rather than on device superiority.

## Case presentation

A seven-year-old boy was brought to our hospital with impaired consciousness (Glasgow Coma Scale score of 10), headache, and vomiting. His perinatal, developmental, and family histories were unremarkable.

**Figure 1:**
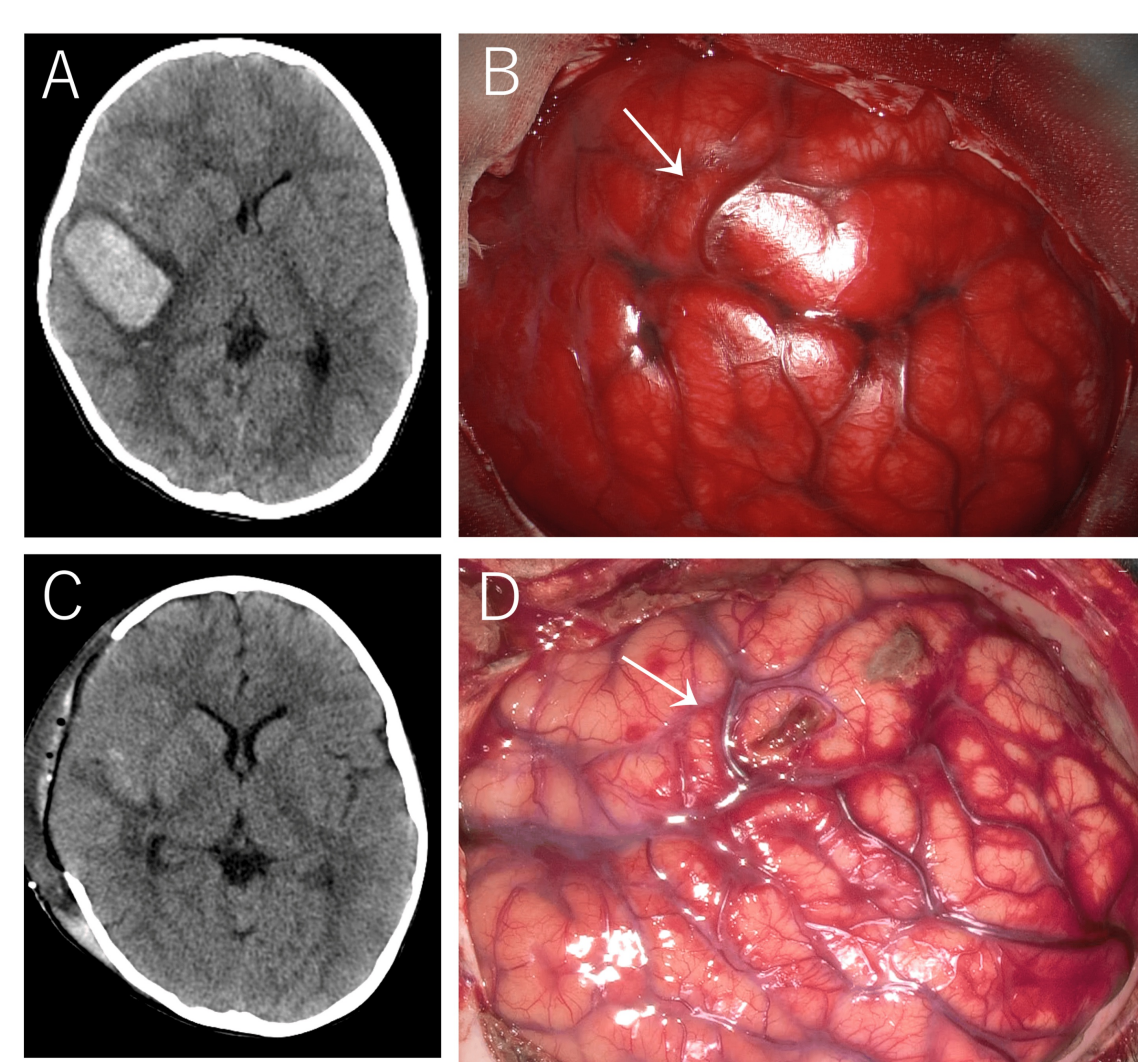
Imaging findings (A) CT at presentation shows a subcortical hemorrhage in the right temporal lobe. (B) Intraoperative photograph immediately before hematoma evacuation shows a bulging, hyperemic brain. A vein anatomically consistent with the vein of Labbé (white arrow), serving as the draining vein of the pial arteriovenous fistula (pAVF), appears engorged and reddish. (C) Postoperative CT demonstrated no new hemorrhage and confirmed the presence of a temporary cranial defect consistent with bone flap removal. (D) During cranioplasty, the vein anatomically consistent with the vein of Labbé (white arrow) appears similar in color to adjacent normal cortical veins.

Computed tomography (CT) revealed a subcortical hemorrhage in the right temporal lobe with sulcal effacement, a hematoma volume of 24 mL, and a midline shift of 4 mm (Figure 1A).

**Figure 2:**
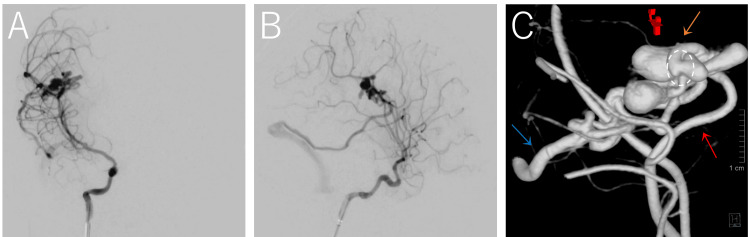
Anteroposterior (A), lateral (B), and three-dimensional reconstructed (C) DSA of the right internal carotid artery demonstrate a pial arteriovenous fistula with multiple venous varices up to 25 mm in size supplied by the superior trunk of the right middle cerebral artery (red arrow) and draining into a vein anatomically consistent with the vein of Labbé (blue arrow). A normal arterial branch (orange arrow) arises proximal to the fistulous point (circle). DSA: digital subtraction angiography

Digital subtraction angiography (DSA) demonstrated a pAVF with multiple venous varices up to 25 mm in size fed by the superior trunk of the right middle cerebral artery (MCA) with venous drainage into a vein anatomically consistent with the vein of Labbé (Figure 2).

The patient underwent emergency decompressive craniectomy with evacuation of the intracerebral hematoma and preservation of the bone flap (Figures 1B, 1C), and elective transarterial embolization (TAE) was subsequently planned on hospital day 7.

**Figure 3:**
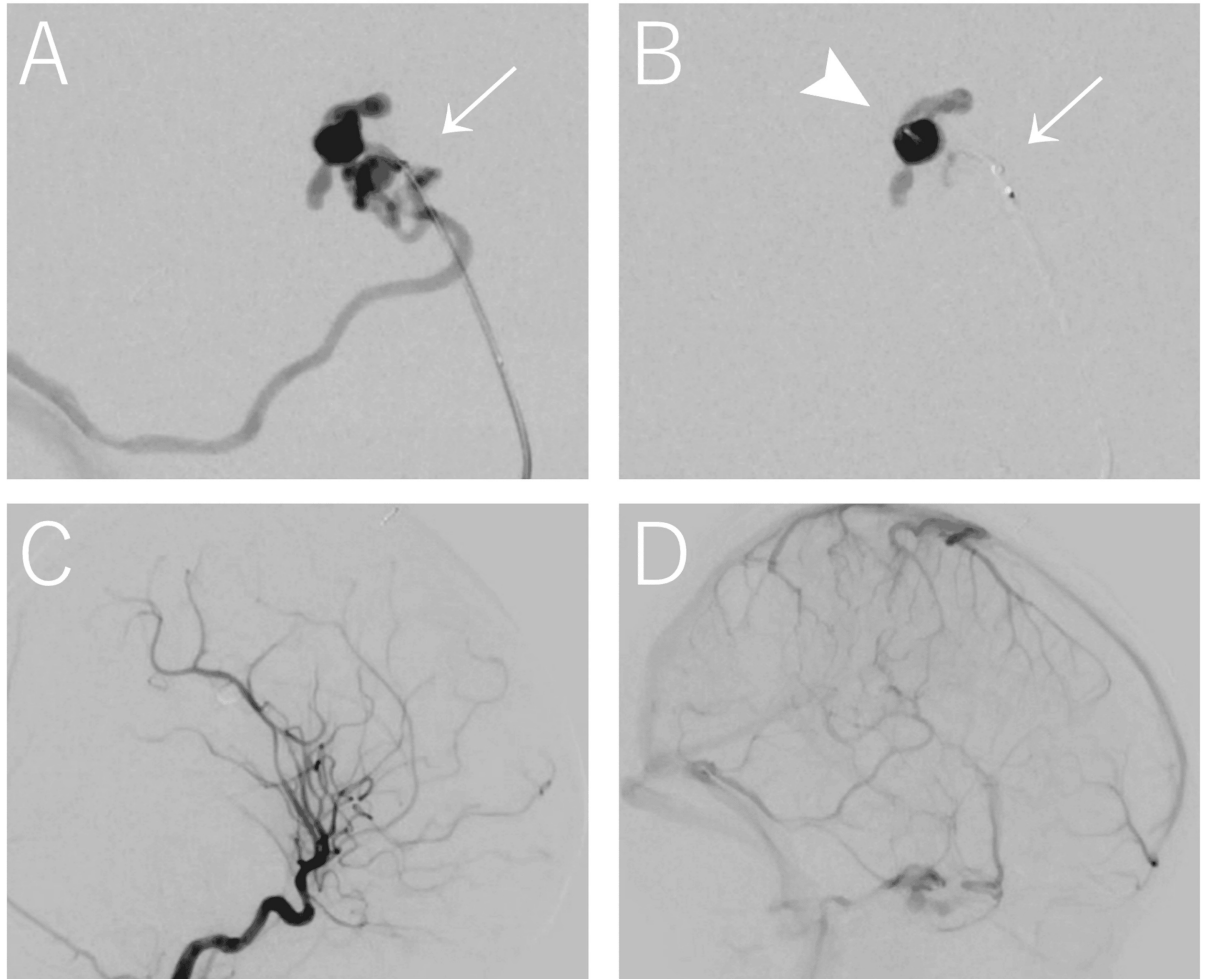
(A) DSA of the superior trunk of the middle cerebral artery demonstrates the fistula and venous varices draining into a vein anatomically consistent with the vein of Labbé, with contrast injected through the AXS Vecta 46 intermediate catheter (white arrow). (B) The fistula and venous varices are occluded with NBCA delivered through an Echelon-10 microcatheter (white arrowhead) supported by the distal access intermediate catheter (white arrow). (C) Post-embolization DSA in the arterial phase shows angiographic obliteration of the fistula. (D) Post-embolization DSA in the venous phase demonstrates preservation of the cortical venous drainage pathway, including the vein anatomically consistent with the vein of Labbé, with restoration of antegrade venous flow. DAS: digital subtraction angiography; NBCA: n-butyl cyanoacrylate

A 5-French ASAHI FUBUKI guiding sheath (Asahi Intecc, Aichi, Japan) was positioned in the high cervical segment of the internal carotid artery via the right femoral artery. The AXS Vecta 46 intermediate catheter (Stryker Neurovascular, Fremont, California, United States) was advanced and positioned approximately 20 mm proximal to the fistula to provide distal support and to allow contrast injection while the microcatheter was in place. A roadmap was then obtained through the distal access intermediate catheter (Figure 3A). Subsequently, an Echelon™-10 Micro Catheter (Medtronic plc, Galway, Ireland) was navigated over a Chikai 14 guidewire (Asahi Intecc) into the distal middle cerebral artery and advanced as close to the fistula as possible. Superselective angiography through the microcatheter demonstrated a normal arterial branch proximal to the fistulous point; therefore, it was advanced beyond this branch and positioned adjacent to the fistula, just proximal to the venous varix.

Embolization was performed using a maximum of 0.5 mL of 60% NBCA injected through the microcatheter, which filled the region near the fistula and venous varices with minimal reflux, and the total injection time was 21 seconds (Figure 3B). Final angiography demonstrated complete shunt disconnection with preservation of the cortical venous drainage pathway (Figure 3C, 3D). The fluoroscopy time was 21 minutes, and the total procedural air kerma was 157 mGy. No procedural complications, including NBCA reflux, venous occlusion, or catheter adhesion, were observed.

The patient required postoperative intensive care unit management for 15 days, after which neurological status gradually improved. Postoperative magnetic resonance imaging showed no signal abnormalities suggestive of acute ischemia. Cranioplasty was performed on hospital day 9. Intraoperatively, the draining vein appeared normally colored, consistent with effective shunt disconnection (Figure 1D, white arrow). The subsequent clinical course was uneventful. At the six-month follow-up, the patient showed no apparent neurological sequelae and was considered to have returned to a functional status comparable to the pre-morbid condition, without cognitive or motor deficits, and had resumed regular attendance at elementary school.

## Discussion

This case illustrates practical considerations in microcatheter-based NBCA embolization for a pediatric high-flow pAVF. In such lesions, precise microcatheter positioning and controlled embolic delivery are essential to avoid reflux, distal migration, or inadvertent compromise of venous drainage [1,4].

Embolic agent selection

NBCA was selected because its rapid polymerization allows decisive shunt disconnection in small-caliber, high-flow fistulous connections [2,3]. Onyx may permit more prolonged injection and controlled penetration in other anatomical settings; however, considerations such as procedural duration and agent-specific characteristics have been variably discussed in the literature [1,4]. Accordingly, embolic selection should be individualized [1,2].

Access strategy and alternatives

Distal access intermediate catheter support was used to enhance microcatheter stability and allow contrast injection with the microcatheter in place [4,5]. Although the use of a distal access intermediate catheter subjectively appeared to improve microcatheter stability during injection, this impression lacks objective validation. Balloon-assisted or coil-assisted flow reduction was not employed in this case due to anatomical considerations and the desire to avoid additional device manipulation in fragile pediatric vessels. These techniques remain valid alternatives depending on the lesion configuration and flow characteristics [4].

Flow control and radiation considerations

Flow reduction was inferred based on the catheter-to-vessel ratio; however, although intermediate catheter occupancy may theoretically influence local flow, no quantitative hemodynamic assessment was performed, and any flow-modulating effect remains speculative. Similarly, although procedural efficiency can influence radiation exposure, this single case cannot establish any reduction in radiation relative to other embolic strategies. Objective radiation metrics are therefore reported without implying causality.

Limitations

This report is limited by its single-case nature, lack of quantitative flow measurements, absence of comparative catheter performance data, incomplete documentation of embolization parameters, and a short-term follow-up.

## Conclusions

Transarterial NBCA embolization through a microcatheter supported by a distal access intermediate catheter achieved angiographic shunt disconnection in this pediatric pAVF. This case report is descriptive and does not establish comparative safety, efficacy, flow-control benefit, or radiation reduction. Further studies with larger cohorts and quantitative assessments are required to clarify the reproducibility and generalizability of this approach.
